# The Story of Bacteria and Their Relation to Health and Disease

**Published:** 1889-12

**Authors:** 


**The Story of Bacteria and their Relation to Health and Disease.** By T. MITCHELL PRUDDEN, M. D. New York and London: G. P. Putnam Sons. 1889.

In a neat little volume of 143 pages, the author gives a short history of Bacteria, their mode of development, and the diseases they produce. It is intended especially for the lay reader, enabling him to form some conception of the modern views of disease and their causative agents. The several chapters are clearly and intelligently written with avoidance of all technical terms, and can be recommended to the unscientific as both useful and interesting. Some points dealing with the prophylaxis of certain infectious and contagious affections, go to make the book of considerable value to those ignorant of the laws of disease.

The press-work is nicely and cleanly done, and is creditable to the publishers. W. C. K.

